# HMGB1 promotes myeloid-derived suppressor cells and renal cell carcinoma immune escape

**DOI:** 10.18632/oncotarget.18796

**Published:** 2017-06-28

**Authors:** Jinfeng Li, Jiajia Sun, Ruiming Rong, Long Li, Wenjun Shang, Dongkui Song, Guiwen Feng, Feifei Luo

**Affiliations:** ^1^ Kidney Transplantation Unit, The First Affiliated Hospital of Zhengzhou University, Zhengzhou, China; ^2^ Department of Urology, Zhongshan Hospital and School of Basic Medical Sciences, Fudan University, Shanghai, China; ^3^ Department of Urology, The First Affiliated Hospital of Zhengzhou University, Zhengzhou, China; ^4^ Department of Digestive Diseases, Huashan Hospital and Biotherapy Research Center, Fudan University, Shanghai, China

**Keywords:** high-mobility group box-1, myeloid-derived suppressor cells, renal cell carcinoma

## Abstract

Despite high immunogenicity and marked presence of immune cells in the RCC(renal cell carcinoma), immunotherapy fails to develop effective anti-tumor immune responses. This is due to the negative regulatory factors in the tumor microenvironment. As the main contributor of immunosuppression, myeloid-derived suppressor cells (MDSCs) inhibited anti-tumor immunity and promoted tumor progression. Meanwhile, it is confirmed that high mobility group box-1 protein (HMGB1) shows a high expression in many solid tumors and HMGB1 with high expression is involved in tumor immune escape. However, the mechanisms linking HMGB1 with tumor immune escape are unclear. The present study aimed to explore whether HMGB1 can promote RCC immune escape by inducing the generation of MDSCs. In this study, Renca mouse model was established and the influence of HMGB1 on MDSCs was investigated by using HMGB1 antibody to downregulate the expression of HMGB1 in tumor-bearing mice. The result showed that with the down-regulation of HMGB1, the tumor growth was inhibited significantly and the mice survival was prolonged greatly. Furthermore, the differentiation and proliferation of MDSCs were inhibited both *in vitro* and *in vivo*, and the inhibition rate showed a positive correlation with the degree of down-regulation of HMGB1. When MDSCs were eliminated with Gr-1 antibody *in vivo*, the ability of the HMGB1 to promote tumor growth was severely impaired. Thus, our findings indicated that HMGB1 might mediate tumor immune escape by promoting MDSCs cell proliferation, which provided a novel theoretical basis for preventing RCC using HMGB1 as the target.

## INTRODUCTION

Immunotherapy develops effective anti-tumor immune responses, which is mainly to mobilize immune system to produce a large number of immune factors or to give exogenous immune factors [1]. As renal cancer cell is characterized as an immunogenic tumor, there is the marked presence of immune cells in the RCC(renal cell carcinoma) tumor tissue [2–4]. However, the efficiency was not high when the renal carcinoma patients were treated with IL-2 and interferon (IFN)-α [5, 6]. In response to this problem, the researchers found that fresh CD8+T lymphocytes isolated from renal cell carcinoma exhibited weak cytotoxicity, whereas these cells showed strong cytotoxicity after they were cultured *in vitro* for a period of time [7, 8]. This implies that the ability of the immune response to control tumor growth is severely inhibited.

Myeloid-derived suppressor cells (MDSCs) are a group of heterogeneous cells derived from bone marrow that have a significant inhibitory effect on immune cell responses. Gradually, the key role of MDSCs in the development of tumors had also been confirmed [9, 10]. Many studies showed that MDSCs was involved in tumor immune escape and promoted tumor progression [11, 12]. On one hand, vascular endothelial growth factor, granulocyte-colony stimulating factor, granulocyte-macrophage colony stimulating factor, IL-6, tumor necrosis factor (TNF)-α, and so forth promote the proliferation and differentiation of bone marrow stromal cells into MDSCs by activating nuclear factor kappa B (NF-κB) and Janus kinase/signal transducers and activators of transcription (JAK/STAT) signal pathway [13–16]. The mediators such as IFN-γ and TGF-β generated by tumor stromal cells and activated T cells can also directly activate MDSCs [17], which help tumor escape from immune surveillance and attack. On the other hand, MDSCs can inhibit the viability of nature killer (NK) cell [18], increase the expression and activity of inducible nitric oxide synthase and arginase1 [19, 20], enhance the secretion of suppressive cytokines (such as TGF-β) [21, 22], and induce the generation of tumor antigen-specific cells [23, 24], further directly or indirectly influencing the proliferation and activation of T cells and inhibiting antitumor immunity. In addition, MDSCs can interact with immunosuppressive M2 tumor-associated macrophage via TGF-β and IL-10, which improve suppressive immune microenvironment [25, 26]. MDSCs have been confirmed as the important cell subset causing tumor immune escape. Therefore, clearing immunosuppressive MDSCs, removing tumor immune tolerance status and mobilizing systemic immune killing function can provide possibly promising strategies for tumor immunotherapy.

High-mobility group box-1 (HMGB1) is a kind of non-histone protein in chromatin, abundant in eukaryotic cell nuclei. Wang et al. reported the involvement of HMGB1 in the pathogenesis of septicopyemia as an important inflammatory factor at first [27]. Lately, some studies demonstrated that HMGB1 was highly expressed in many solid tumors, such as melanoma, nasopharynx cancer, breast cancer, colorectal cancer, cervical cancer and bladder cancer [28–32]. A previous study also indicated that HMGB1 was highly expressed in RCC, and the expression level showed a positive correlation with cancer bearing, metastasis, and clinical staging and grading [33]. By inhibiting caspase activity, increasing NF-κB activity and upregulating the cellular inhibitor of apoptosis protein-2, HMGB1 can inhibit apoptosis of tumor cells, thus promoting the occurrence and development of tumor [34]. Also, Liu et al. proved that the high expression of HMGB1 could promote regulatory T (Treg) cells to secrete IL-10 and weaken the antitumor effect of CD8+ T cells [35]. As a multifunctional cytokine, HMGB1 plays a key role in tumor formation, metastasis and immune escape [36].

It has been confirmed that the aggregation of MDSCs at tumor site helps the immune escape of tumor cells. Meanwhile, HMGB1 is significantly highly expressed in tumor tissue. However, the relationship between HMGB1 and MDSCs in tumor immune escape is studied rarely [37]. In this study, by *in vitro* and *in vivo* experiments, we indicated that HMGB1 mediated tumor immune escape by promoting MDSC cell proliferation.

## RESULTS

### Downregulation of HMGB1 slows the progression of RCC

**Figure 1:**
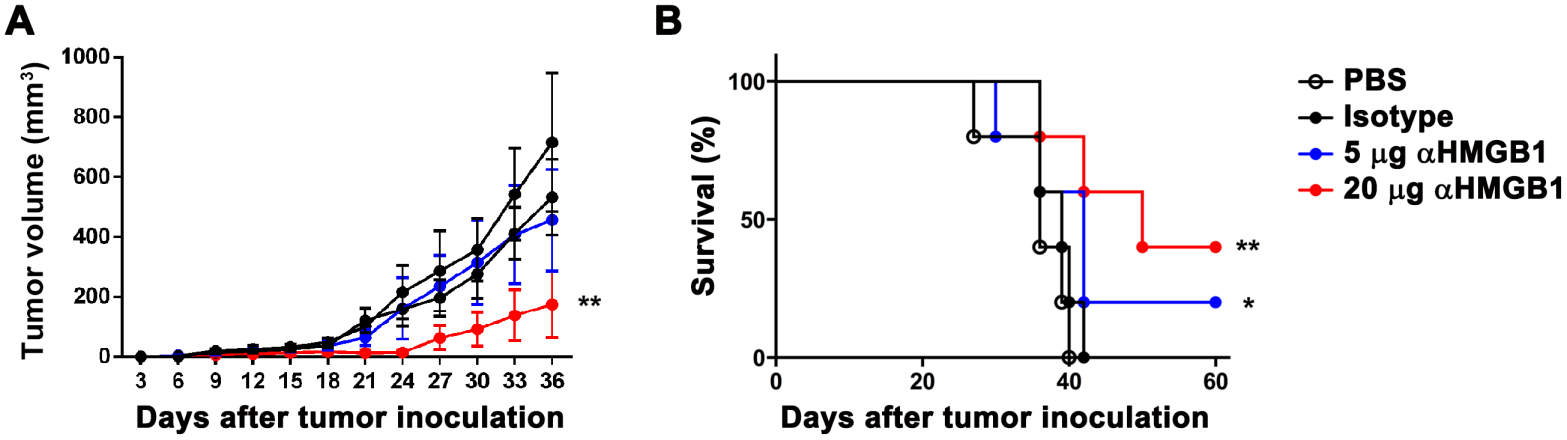
Dose-dependent effect of αHMGB1 on tumor remission and host survival BALB/c mice were implanted with Renca tumor cells on day 0 and injected with *i.p*. PBS, isotype antibody, or different doses of αHMGB1Ab every 3 days. Tumor development **(A)** and mortality **(B)** was monitored regularly and statistically analyzed using a log-rank test (n=8). Data are mean±SEM. Representative results of one of three independent experiments. *P<0.05, **P<0.01.

To study the effect of HMGB1 on cancer, the influence of two doses (5 and 20 μg) of αHMGB1 antibody(Ab) on the Renca-bearing mice was first detected. Renca tumor cells were subcutaneously injected into the BALB/c mice. Meanwhile, the mice were intraperitoneally injected with 100 μL PBS, 20 μg mouse IgG2b isotype Ab, 5 μg or 20 μg αHMGB1Ab every 3 days for total seven times. The tumor growth and mice survival were monitored periodically. Compared with the two control groups, the tumor growth was inhibited significantly and the host survival was prolonged greatly in αHMGB1Ab-treated groups, which displayed a dose-dependent manner (Figure 1). Our results demonstrated that blockage of HMGB1 inhibited RCC progression.

### HMGB1 does not mediate the pro-tumor effect by directly inhibiting the proliferation of T cells and B cells

**Figure 2:**
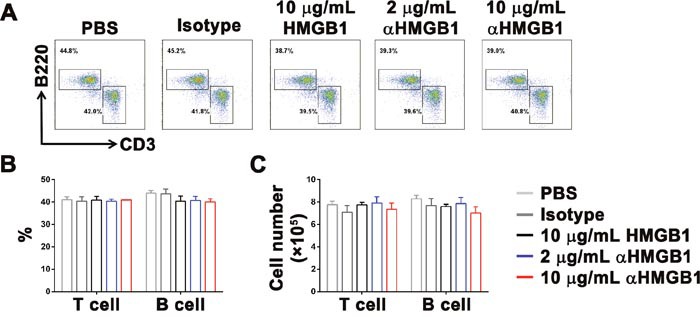
HMGB1 does not influence the development and proliferation of T cells and B cells Bone marrow cells were stimulated with HMGB1 (10 μg/mL), αHMGB1Ab (2 μg/mL), αHMGB1Ab (10 μg/mL), PBS (10 μg/mL) or isotype control Ab (10 μg/mL) *in vitro*. **(A, B** and **C)** Two days later, the percentage and cell number of T and B cells were detected by FACS and trypan blue assay. Data are mean±SEM. Representative results of one of three independent experiments.

Then, we investigated the mechanism underlying the promotion of tumor growth in the Renca-bearing mice model by HMGB1. One hypothesis was that HMGB1 directly inhibited the differentiation and proliferation of T cells and B cells, and then caused tumor cell to escape from monitoring and killing by the immune system. Therefore, bone marrow cells were isolated from Renca-bearing mice by MACS and stimulated with PBS, 10 μg/mL isotype control Ab, 10 μg/mL HMGB1, 2 μg/mL or 10 μg/mL αHMGB1Ab *in vitro*. Two days later, the percentage and cell number of T and B cells were detected by FACS and trypan blue assay. The data showed that there was no difference in the percentage and cell number T and B cells (Figure 2). Furthermore, the Renca-bearing mice were intraperitoneally injected with 100 μL PBS, 20 μg mouse IgG2b isotype Ab, 5 μg or 20 μg αHMGB1 Ab every 3 days for total seven times. One day after the final treatment, the expression levels of CD3 and B220 in spleens were analyzed by FACS. As shown in Figure 4A, no significant difference was observed between the αHMGB1 Ab-treated mice and the control mice. The above *in vitro* and *in vivo* data indicated that HMGB1 couldn't mediate the occurrence and development of tumor by directly inhibiting the differentiation and proliferation of T cells and B cells.

**Figure 3:**
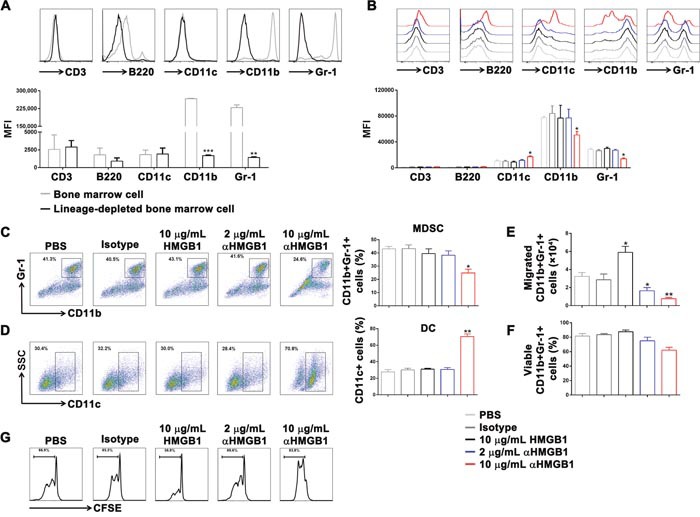
Downregulation of HMGB1 induced differentiation of DCs and inhibited differentiation of MDSCs *in vitro* Lineage-depleted bone marrow cells were purified from tumor-bearing mice using the MACS. Then, HMGB1 (10 μg/mL), αHMGB1Ab (2 μg/mL), αHMGB1Ab (10 μg/mL), PBS (10 μg/mL) or isotype control Ab (10 μg/mL) was added into lineage-depleted bone marrow cells and incubated for 5 days. **(A)** The expression of CD3, B220, CD11c, CD11b, and Gr-1 on the surface of bone marrow cells and lineage-depleted bone marrow cells was measured by flow cytometry. **(B)** The expression of CD3, B220, CD11c, CD11b, and Gr-1 of lineage-depleted bone marrow cells from each group was measured by flow cytometry. **(C and D)** The CD11b+Gr-1+ MDSCs ratio and CD11c+ DCs ratio were analyzed by FACS. **(E)** The migrated cell number of CD11b+Gr-1+ MDSCs from each group was determined by FACS and transwell assay. **(F)** The percentage of viable CD11b+Gr-1+ MDSCs from each group was analyzed by FACS and trypan blue assay. **(G)** The CD11b+Gr-1+ MDSCs from each group were purified and co-cultured with CFSE-labeling T cells. After 4 days, the proliferation of T cells was detected by flow cytometry. Data are mean±SEM. Representative results of one of three independent experiments. *P<0.05, **P<0.01.

After that, different doses of αHMGB1Ab were used to induce differentiation of lineage-depleted bone marrow cells *in vitro*. The results showed that in the 10 μg/mL αHMGB1Ab group, the expressions of CD3 and B220 were only slightly increased; however, no improvement in the differentiation of T cells and B cells in the 2 μg/mL HMGB1 and control groups was observed (Figure 3A).

### Downregulation of HMGB1 induces CD11c+ dendritic cells and inhibits CD11b+ Gr-1+ MDSCs

Next, in order to study the influence of HMGB1 on MDSC differentiation, lineage-depleted bone marrow cells were sorted from Renca-bearing mice by MACS, which didn't express CD3, B220, CD11c, CD11b, and Gr-1 (Figure 3A). Then, PBS (100 μL), isotype control Ab (10 μg/mL), HMGB1 (10 μg/mL), αHMGB1Ab (2 μg/mL), or αHMGB1Ab (10 μg/mL) with IL-6 and GM-CSF was added into lineage-depleted bone marrow cells and incubated for 5 days. The flow cytometry results showed that no significant difference was observed in the 10 μg/mL HMGB1 group and the 2 μg/mL αHMGB1Ab group, compared with the control group, whereas 10 μg/mL αHMGB1Ab significantly increased the expression of CD11c and decreased the expression of CD11b and Gr-1 (Figure 3B). Furthermore, 10 μg/mL HMGB1 increased significantly the frequency of CD11c+ dendritic cells (DC) (70.7%) and decreased greatly the frequency of CD11b+Gr-1+ MDSC (24.9%) (Figure 3C, 3D). Meanwhile, the migration and viability of CD11b+Gr-1+ MDSC were detected by transwell and trypan blue assay. Compared with control groups, 10 μg/mL αHMGB1Ab could significantly inhibit the migration of MDSC, and slightly decrease the activation of MDSC (Figure 3E, 3F). After that, these CD11b+Gr-1+ MDSCs were co-cultured with CFSE-labeling T cells at a ratio of 1:5. Four days later, the proliferation of T cells were determined by FACS. As shown in Figure 3G, the proliferation of T cells were greatly enhanced when co-cultured with 10 μg/mL αHMGB1Ab-pretreated MDSC, compared with other groups. Thus, the blockage of HMGB1 could induce the production of DC and inhibiting the differentiation, migration and suppressive functions of MDSCs.

### HMGB1 promotes renal cell carcinoma immune escape by inducing MDSC proliferation in the Renca-bearing mice

**Figure 4:**
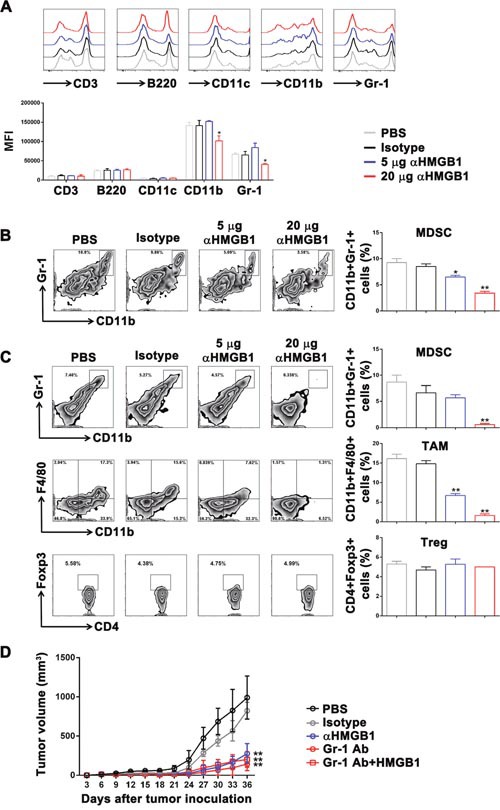
Downregulation of HMGB1 inhibited MDSCs proliferation in the Renca tumor-bearing mice The Renca-bearing mice of each group (n=5) were intraperitoneally injected with 5 and 20 μg αHMGB1Ab, 100 μL of PBS and 20 μg isotype control Ab, respectively. Splenocytes and tumors were isolated from tumor-bearing mice. **(A)** The surface expression of CD3, B220, CD11c, CD11b, and Gr-1 on splenocytes was detected by flow cytometry. **(B)** The splenic MDSCs ratio was measured by FACS. **(C)** The MDSCs, TAM and Treg ratio at tumor site was measured by FACS. **(D)** The Renca-bearing mice of each group were intraperitoneally injected with 100 μL of PBS, 20 μg isotype control Ab, 20 μg αHMGB1Ab, 10 μg αGr-1, and 10 μg αGr-1Ab plus 20 μg HMGB1 every 3 days from day 0. Tumor development and mortality was monitored regularly and statistically analyzed using a log-rank test (n=5). Data are mean±SEM. Representative results of one of three independent experiments. *P<0.05, **P<0.01.

It has been confirmed that MDSC participates in tumor immune tolerance, and the downregulation of HMGB1 in bone marrow cells *in vitro* could significantly inhibit the differentiation of MDSCs. Therefore, we further investigated whether HMGB1 could promote renal cell carcinoma by enhancing the proliferation of MDSCs. The Renca-bearing mice were intraperitoneally injected with 100 μL PBS, 20 μg isotype, 5 or 20 μg αHMGB1Ab every 3 days for total seven times (*n*=5). One day after the final treatment, mice were sacrificed, and the expression of surface markers in spleens was detected using flow cytometry. It was found that 20 μg αHMGB1Ab did not change the expression level of CD11c in the mice model, but it significantly decreased the expression of CD11b and Gr-1 (Figure 4A). Besides, the frequency of CD11b+Gr-1+ MDSC in the spleens of the tumor-bearing mice was significantly decreased (Figure 4B). At the tumor sites, the frequency of MDSCs and TAMs were decreased greatly in 20 μg αHMGB1Ab group, but not the frequency of Treg, compared with those in other control groups (Figure 4C). Also, the Figure 1 results suggested that the growth of subcutaneous tumor cells treated with 20 μg αHMGB1Ab was significantly inhibited, and the survival rate also increased. Therefore, it could be deduced that HMGB1 could realize renal cell carcinoma immune escape by inducing the proliferation and differentiation of MDSCs. In order to further confirm the above conclusion, Renca-bearing mice were intraperitoneally injected Gr-1 antibody to deplete MDSC and treated with or without 20 μg HMGB1 every 3 days for total seven times (*n*=5). As expected, compared with control groups, the tumor growth was significantly delayed after MDSC depletion, which was similar with that in 20 μg αHMGB1Ab group. And even exogenous HMGB1 couldn't recover the delayed tumor growth by Gr-1 antibody (Figure 4D). Thus, HMGB1 might promote renal cell carcinoma mainly via MDSC. Furthermore, compared with the control groups, in the 5 μg HMGB1Ab group, the expression of CD11b and Gr-1 as well as CD11b+Gr-1+MDSC ratio in spleens and tumors did not significantly change (Figure 4B, 4C). Our data demonstrated that the effect of HMGB1 on MDSC was dose dependent, and only high dose of HMGB1 could inhibit the differentiation and proliferation of MDSCs and mobilize the body's immune activity to kill tumor.

## DISCUSSION

As an important contributor of immunosuppression, MDSCs promote tumor immune escape by various immunologic mechanisms [18–26, 38, 39]. This study found that, by down-regulating HMGB1 secretion and expression in the Renca-bearing mice, HMGB1Ab induced a significant decrease in the proliferation and differentiation of MDSCs in bone marrow cells. Meanwhile, the tumor growth in the mice was also inhibited. HMGB1 could potentially regulate the amplification and involvement of MDSCs in tumor immune escape.

Currently, HMGB1 Ab, HMGB1 antagonist, HMGB1 inhibitor, and nucleic acid technology inhibit expression, secretion, and signal transduction of HMGB1 in tumor cells, which plays a key role in anti-inflammation therapy [40–43]. However, these methods are still needed further verification *in vitro/in vivo*. Zitvogel [44] proved that dead tumor cells could release HMGB1, thus activating tumor-specific T cell immunity, and inducing antitumor effect via toll-like receptor 4 stimulating dendritic cells. Other studies [45–48] reported that HMGB1 could also induce the maturation of immature dendritic cells and initiate adaptive immune response as an *in vitro* signal. Tracey group [49] found that dendritic cells activated by HMGB1 could stimulate the activation and proliferation of T cells as helper T lymphocytes. However, the present study showed that by increasing the immunosuppression of MDSCs, HMGB1 successfully promoted the proliferation and development of renal cell carcinoma cells. The results indicated that HMGB1 signal generated two different immune effects during tumor development. The possible reason was that HMGB1 mediated different signaling pathways and further caused dual biological effects [50]. Such dual regulation mechanism provided greater challenges for immunotherapy using HMGB1 as the target.

In the present study, HMGB1Ab was used to down-regulate HMGB1 secretion in the tumor-bearing mice. The result indicated that the proliferation of mature T lymphocytes and B lymphocytes in bone marrow cells was not directly influenced. It suggested that highly expressed HMGB1 did not mediate the proliferation of tumor cells by directly inhibiting adaptive immune response. Furthermore, HMGB1Ab could induce the differentiation of mature DC cells, inhibit the differentiation of MDSCs, and indirectly lead to a slightly high expression of CD3 and B220 *in vitro*. This verified that HMGB1 promoted the differentiation and amplification of MDSCs primarily and inhibited innate and adaptive response by activated MDSCs to escape body's monitoring and killing. However, the present results did not exclude other effects of HMGB1 in tumor immune escape. For example, HMGB1 could activate the JAK/STAT pathway, decrease tumor cell apoptosis, promote cell cycle, and induce resistance and immune escape in tumor cells [51, 52]. Besides, Liu et al. [36] also proved that the high expression of HMGB1 could promote Treg cells to secrete IL-10 and weaken antitumor effect of CD8+ T cells.

Furthermore, it was noted that a low dose of HMGB1Ab did not inhibit the proliferation and differentiation of MDSCs significantly in the *in vitro* experiment (Figures 2 and 3). Compared with the high dose, the inducing effects of the low dose of HMGB1Ab on tumor remission and host survival were poor. It was because the tumor cells and activated immune cells, such as macrophages, dendritic cells, and NK cells, could actively secrete HMGB1 [53, 54]. The low dose of HMGB1 Ab could not significantly change the expression and secretion of HMGB1 in mouse model and influence the inducing effect of HMGB1 on the amplification of MDSCs. The result indicated that only HMGB1 in the mouse model was down-regulated to a certain level; the role of HMGB1 in regulating MDSC proliferation and indirectly mediating immune escape could be eliminated.

This study reported the tumor-inhibiting effect of HMGB1Ab in the Renca-bearing mouse model. More importantly, the study indicated that HMGB1 could mediate tumor immune escape by improving MDSC proliferation, which provided a novel theoretical basis for antitumor therapy using HMGB1 as the target. However, the detailed mechanism underlying the regulation of MDSC proliferation by HMGB1 is not clear. The dual immunological effect of HMGB1 on tumor therapy is also unexploited, which needs further exploration.

## MATERIALS AND METHODS

### Mice and cell line

Male BALB/c mice (6- 8 weeks) were purchased from SLAC Laboratory Animal Co., Ltd. (Shanghai, China) and raised in specific-pathogen-free animal room. All the animal experiments complied with the Care and Use of Laboratory Animals (No. 55 issued by the Ministry of Health, People's Republic of China on January 25, 1998).

The Renca murine RCC cell line was purchased from TW Reagent (Shanghai, China). The cells were incubated in RPMI 1640 (Gibco) containing 10% fetal bovine serum (Gibco), 0.1mM nonessential amino acid (Gibco), 1mM sodium pyruvate (Gibco), 2mM L-glutamine (Gibco), and 1% antibiotic antimycotic solution (Gibco) under 37°C, 5% CO_2_, and saturated humidity.

### Tumor model

The tumor-bearing mouse model was established by subcutaneously injecting the tumor cells, which was convenient to measure the tumor size. Renca cells under logarithmic phase were collected, and the density was adjusted at 2×10^7^/mL. Pentobarbital sodium (200 μL) was intraperitoneally injected into the BALB/c mice. After general anesthesia, the BALB/c mice were subcutaneously injected with the cell suspension (100 μL) at the left groin (2×10^6^/mouse). The tumor volume was observed and detected every 3 days. The tumor area was measured using a digital caliper and calculated using the formula: (length X width^2^)/2.

### Isolation of lineage-depleted bone marrow cells *in vitro*

One week post tumor implantation, the mice were sacrificed, the thigh bone and tibia were peeled, and the bone marrow cells were collected using an injection syringe. The cells were sorted using the Lineage Cell Depletion MACS (magnetic activated cell sorter) kit (MiltenyiBiotec) following the manufacturer instructions, and the lineage-depleted bone marrow cells were obtained *in vitro* with a cell yield rate of 3%. The levels of CD3, B220, CD11c, CD11b, and Gr-1 were detected using flow cytometry before and after sorting.

### HMGB1 transforming lineage-depleted bone marrow cells *in vitro*

The cell concentration of sorted lineage-depleted bone marrow cells was adjusted at 1×10^6^/mL, and the cells were added into a 24-well plate (1 mL/well,20 wells are used). The wells with cells were randomly divided into five groups: phosphate-buffered saline (PBS) (Hyclone), 10 μg/mL HMGB1 (Biolegend), 10 μg/mL Mouse IgG2b, κ Isotype (Biolegend), and 2 and 10 μg/mL HMGB1 antibody (Biolegend) were addeded, respectively. All cells were incubated with 20 ng/mL IL-6 (R&D) and GM-CSF (R&D) for 5 days, and the levels of CD3, B220, CD11c, CD11b and Gr-1 were detected using flow cytometry.

### HMGB1 inducing experiment in the tumor-bearing mice *in vivo*

During 0-21 days of tumor inoculation, the above tumor-bearing mouse were injected with 100 μL PBS (Hyclone), 20 μg/mL Mouse IgG2b, κ Isotype (Biolegend), 5 or20 μg/mL HMGB1 antibody (Biolegend) every 3 days for total seven times, respectively. And the subcutaneous tumor sizes were measured every 3 days. At day 21, the mice were sacrificed, spleen and tumor tissues were extracted, and the single cell suspension was prepared. The levels of CD3, B220, CD11c, CD11b, and Gr-1 in spleens and the levels of CD11b, Gr-1, F4/80, CD4 and Foxp3 in tumors were detected using flow cytometry.

### Flow cytometry

Lineage-depleted bone marrow cells, tumor cells and spleen cells before and after treatment were collected and re-suspended in 40 μL PBS (Hyclone). Then, 10 μL Fc block (eBioscience) was added to the cell suspension to block Fc receptor for 15 min at 4°C. Next, 50 μL of fluorescence-labeled antibody was added for 30 min at 4°C, washed with PBS (2 mL) twice, and re-suspended in PBS (300 μL). Gallios flow cytometer (Beckman Coulter, USA) was used to detect cell phenotype. Fluorescence-linked anti-CD3 (Clone: 145-2C11), anti-B220 (Clone: RA3-6B2), anti-CD11c (Clone: N418), anti-CD11b (Clone: M1/70), anti-Gr-1 (Clone: RB6-8C5), anti-F4/80 (Clone: BM8), anti-CD4 (Clone: GK1.5), and anti-Foxp3 (Clone: FJK-16s) (eBioscience) were used to stain surface markers of T cells, B cells, CD11c+ dendritic cells, CD11b+Gr-1+ MDSC cells, CD11b+F4/80+ TAM cells, CD4 +Foxp3+ Treg cells.

### Statistical analysis

GraphPad Prism version 6.0 was used to analyze the data. Single factor *t* test (comparison between groups) and one-way analysis of variance (ANOVA) test (comparison among groups) were used to analyze the *in vitro* data. Mann-Whitney *U* test and ANOVA test were used to analyze the *in vivo* data. A *P* value <0.05 (bilateral) was considered statistically significant.
